# Autoimmune Thyroid Diseases and Microscopic Colitis: A Nationwide Matched Case–Control Study in Sweden

**DOI:** 10.1210/clinem/dgaf140

**Published:** 2025-03-05

**Authors:** David Bergman, Xiaoying Kang, Jiangwei Sun, Fahim Ebrahimi, Jonas F Ludvigsson

**Affiliations:** Department of Medical Epidemiology and Biostatistics, Karolinska Institutet, Stockholm 171 65, Sweden; Department of Medical Epidemiology and Biostatistics, Karolinska Institutet, Stockholm 171 65, Sweden; Department of Neurology, Yale School of Medicine, New Haven, CT 06510, USA; Department of Medical Epidemiology and Biostatistics, Karolinska Institutet, Stockholm 171 65, Sweden; Department of Medical Epidemiology and Biostatistics, Karolinska Institutet, Stockholm 171 65, Sweden; Department of Gastroenterology and Hepatology, Clarunis University Center for Gastrointestinal and Liver Diseases, Basel 4031, Switzerland; Department of Medical Epidemiology and Biostatistics, Karolinska Institutet, Stockholm 171 65, Sweden; Department of Pediatrics, Örebro University Hospital, Örebro 701 85, Sweden; Department of Medicine, Columbia University College of Physicians and Surgeons, New York, NY 10032, USA

**Keywords:** Hashimoto thyroiditis, Graves disease, microscopic colitis, epidemiology

## Abstract

**Context:**

Microscopic colitis (MC), comprising collagenous colitis (CC) and lymphocytic colitis (LC), is an inflammatory condition of the colon, characterized by watery diarrhea. Previous studies suggest an association between autoimmune thyroid diseases (AITDs) (Hashimoto thyroiditis and Graves disease) and MC; however, large-scale histology studies are lacking.

**Objective:**

To assess the association between AITDs and future onset of MC.

**Methods:**

We conducted a nationwide, matched case–control study. Patients with biopsy-confirmed MC diagnosed between 2006 and 2017 were identified through the population-based histopathology cohort ESPRESSO. Data on AITDs and covariates were retrieved from Swedish national health care registers. Odds ratios (ORs) for MC associated with prior AITDs were estimated using conditional logistic regression. Sibling comparisons were performed to minimize shared genetic and environmental confounding.

**Results:**

Among 10 301 MC cases and 48 712 controls, AITDs were significantly more prevalent in MC patients (12.0%) than in controls (7.8%), yielding an adjusted OR of 1.65 (95% CI 1.54-1.77). This association was attenuated but remained significant in sibling analyses (OR 1.26; 95% CI 1.11-1.43) The association was stronger in patients diagnosed with MC before age 50 (OR 2.41; 95% CI 2.02-2.89). Subgroup analyses revealed no difference between CC and LC or between sexes.

**Conclusion:**

Individuals with AITDs are at an increased risk of developing MC. That this association was robust across various subgroups may be indicative of shared underlying mechanisms. Our findings highlight the importance of being vigilant of gastrointestinal symptoms in patients with AITDs and that patients with persistent symptoms despite achieving euthyroidism should be evaluated for MC.

Microscopic colitis (MC) is an inflammatory condition of the colon (1). The disease is characterized by watery diarrhea (2) and typically affects middle-aged women (3). MC is subdivided into collagenous colitis (CC) and lymphocytic colitis (LC) (4), and the distinction between these 2 entities is based on histopathological findings in a biopsy from the colon. Partly due to this rather complicated diagnostic procedure, incidence rates have historically been low, reflecting an insufficient awareness of the disease. However, incidence rates of MC (3, 5, 6) now rival those of ulcerative colitis and Crohn disease (7).

There is a known association between MC and autoimmune diseases (8-10). Given its high prevalence in women and its response to corticosteroids, it has been suggested that MC, itself, is an autoimmune condition. To date, however, no specific autoantibody has been identified in MC. While there are previous reports on an association between autoimmune thyroid disease (AITD) (ie, Hashimoto thyroiditis [HT] and Graves disease [GD]) and MC, most of these studies have been cross-sectional and questionnaire-based (11-14), and, to our knowledge, none have detailed the association in its subtypes (9) or corrected for multiple testing (9, 13). Our group has previously confirmed the concordance between a colon biopsy indicating MC and the clinical diagnosis (15). In that validation study, we found that 8% of patients had a diagnosis of HT (15).

Due to the findings of the aforementioned studies, similar inflammatory characteristics of AITDs (16, 17) and MC (18) and the fact that patients with HT have been found to have an increased number of intraepithelial lymphocytes in the colonic epithelium (19) (a hallmark of MC) and that patients with GD have been found to have alterations in their gut microbiome (20), we hypothesized an association between AITDs with future MC.

Hence, we used data from a nationwide histopathology cohort in Sweden to explore this association. Added knowledge in this field could serve as a foundation for future mechanistic studies and to improve awareness of MC in patients with AITDs with gastrointestinal symptoms.

## Materials and Methods

### Setting

Swedish health care is mainly funded by revenue from income tax and designed to provide all citizens with equal access to health care. Data analyzed in this study originate from nationwide health care registers as well as population registers maintained by Swedish government agencies. Linkage between these registers is made possible by all Swedish citizens having a unique personal identity number (21).

### Data Sources

Data on disease diagnoses, exclusion criteria, and covariates were retrieved from the Swedish National Patient Register (NPR) (22). This register stores information on International Classification of Diseases codes and codes designating medical procedures (surgeries). The register attained nationwide coverage for inpatient care in 1987, and since 2001 it also contains data from specialized outpatient care. Information on dispensed medications was gathered from the Prescribed Drug Register (PDR) (23), which, since July 1, 2005, has a virtually complete coverage of dispensed medications. Matched controls were randomly selected from the Total Population Register (24), which, since 1968, maintains records on personal identity number, migration, vital status, and residence of all Swedish citizens. Full siblings of patients with MC were identified from the Multigeneration Register, a subset of the Total Population Register, keeping records of family relations (siblings, parents) of all Swedish citizens. Data on MC was originally retrieved from all of Sweden's 28 regional pathology registers (25). Level of education, used as a proxy for socioeconomic status, was obtained from the Longitudinal Integrated Database for Health Insurance and Labor Market Studies (26). This register holds data on educational attainment on >98% of Swedes aged 25-64 years since 1990. For analytical purposes, we imputed missing education with the highest educational level of the parents and categorized the imputed education into compulsory school (≤9 years), upper secondary school (10-12 years), college (≥13 years), and missing (if parental education was missing as well).

### Design and Study Population

**Figure 1.**
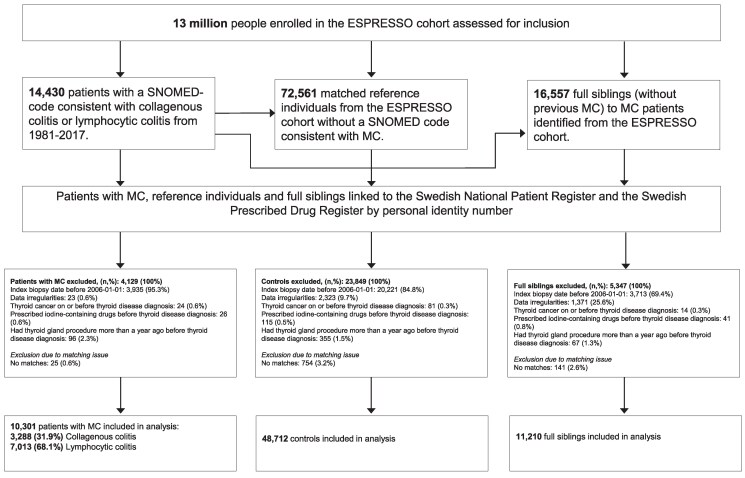
Flowchart of inclusion of patients with biopsy-confirmed microscopic colitis in the ESPRESSO histopathology cohort, siblings and matched general population reference individuals from the Swedish total population register 2006-2017. Abbreviation: ESPRESSO, Epidemiology Strengthened by Histopathology Reports.

Leveraging the nationwide ESPRESSO (Epidemiology Strengthened by histoPathology Reports in Sweden) cohort (25) of 2.1 million unique individuals with 6.1 million gastrointestinal biopsy records, we designed a population-based matched case–control study. All patients with biopsy-confirmed MC (cases) diagnosed between 2006 and 2017 were included in the study (Fig. 1). We chose 2006 as a starting point to enable exclusions and definition of exposures based on data from the PDR. Two separate groups were compared to (1) general controls: MC-free individuals matched from the general population at the time of matching (index date) on age, sex, calendar year, and county of residence; and (2) sibling controls: all MC-free full siblings of the patients with MC.

### Outcome

The outcome variable was biopsy-confirmed MC ascertained from the ESPRESSO study, entailing both CC (per SnoMed code “M40600”) and LC (per SnoMed code “M47170”). Compared to the clinical diagnosis of MC, our histological diagnosis has been reported as having a positive predictive value of 95% (15). Moreover, in the same validation study, we found that 96% of patients with a histopathological diagnosis of MC experienced diarrhea and that 8% had a concomitant diagnosis of hypothyroidism.

### Exposure

Pre-MC/index diagnosis of AITD, defined as either HT (hypothyroidism) or GD (hyperthyroidism), was analyzed as exposure variable. Data on HT was extracted from the PDR and NPR based on a history of prescribed levothyroxine or iothyronine and no earlier record of GD (Table S1 (27)) (28, 29), while data on GD were ascertained from the NPR per relevant International Classification of Diseases codes. For patients converted from HT to GD, date of exposure was defined as the date of HT diagnosis. The patients identified with AITD were then filtered to exclude those with thyroid cancer before the AITD diagnosis, prescribed iodine-containing drugs before the AITD diagnosis, or received thyroid gland procedure more than a year before the AITD diagnosis (Fig. 1; Table S1 (27)), as these procedures may indicate thyroid disease other than our exposures of interest.

### Covariates

Covariates adjusted for in the statistical models include age at index date, sex, birth country (Nordic countries or not), educational level, and a binary variable indicating prevalence of any of 6 confounding autoimmune comorbidities, namely celiac disease, inflammatory bowel disease, asthma, rheumatoid arthritis, type 1 diabetes, and psoriasis (Table S2 (27)). Notably, we included only those autoimmune conditions diagnosed before exposure among the exposed and those before the index date among unexposed individuals as prevalent comorbidities for adjustment. The number of hospital visits (inpatient and outpatient) recorded between 3 years and 6 months before the index date was also computed and categorized into 4 groups per quantile to adjust for surveillance bias in a sensitivity analysis.

### Statistical Analysis

Sample characteristics were described for MC cases vs their population and sibling controls separately. The overall association of MC with prior diagnosis of AITD was estimated as odds ratios (ORs) and 95% CIs via conditional logistic regression, using the clogit function in the R Survival package. As illustrated in Fig. 1, we compared all MC cases diagnosed 2006-2017 to their population controls for the main analysis purpose, adjusting the matching-set identifier as well as birth country, education, and prevalent autoimmune comorbidities as covariates. In a sensitivity analysis, to control for surveillance bias, all study participants with a follow-up less than 1 year were excluded. To account for unmeasured confounding shared between full siblings such as shared genetics and early-life environmental factors, we performed an analysis using sibling controls for comparison, entering the family IDs as stratum and controlling for age at index date, sex, education, and prevalent autoimmune comorbidities as covariates. To minimize misclassification of exposure due to the potential delay of the registration or diagnosis of AITD and to more stringently control for surveillance bias, a sensitivity analysis of MC cases and their population controls who had at least 1 hospital visit during a time window of 3 years to 6 months prior to the index date was implemented using the same model specification as main analysis, including the categorized number of hospital visits as an additional covariate. Difference in associations by MC subtype, age group (categorized at around the first and third quantiles of age at index date), sex, or autoimmune comorbidities (dichotomized based on the prevalence of any of the 6 selected conditions) was explored in subgroup analyses. A 2-sided α < .05 was used to determine statistical significance.

All analyses were performed in R software (version 4.3.1). This study was approved by the Regional Ethics Committee, Stockholm, Sweden (Protocol no 2014/1287-31/4, 2018/972-32 and 2022-05774-02).

## Results

### Characteristics of Population Analysis Sample

A total of 10 301 cases with biopsy-confirmed MC diagnosis (32% CC and 68% LC) and 48 712 matched population controls were included in the study (Fig. 1 and Table 1). Due to matching, cases and controls were of similar age distribution (median [interquartile range] in years: 63.8 [50.6-73.1] for cases and 63.1 [49.9-72.3] for controls) and both comprised more females (71.7%). No prominent difference in educational attainment at index date was observed between the 2 groups. As expected, comorbidities including several autoimmune conditions, such as inflammatory bowel disease, celiac disease, asthma, psoriasis, rheumatoid arthritis, and type 1 diabetes affected patients with MC more frequently, yielding a more than doubled prevalence of any of the 6 autoimmune conditions in cases (18.5%) vs controls (7.9%). AITD, predominantly HT (>90%), was also more commonly diagnosed before the index date among patients with MC (12.0%) than controls (7.8%). In contrast to those in the control group, patients with AITD in MC cases had a slightly younger age at AITD diagnosis (median [interquartile range] in years: 61.1 [49.8-70.1] in cases and 62.4 [52.9-70.7] for controls).

**Table 1.** Characteristics of patients with microscopic colitis (cases) diagnosed between 2006 and 2017 and their matched population controls

	MC cases			Population controls
	Total	CC	LC	
Total (%)	10 301 (100)	3288 (100)	7013 (100)	48 712 (100)
Age at index date, median (IQR)	63.8 (50.6-73.1)	65.9 (56.1-74.5)	62.4 (48.0-72.2)	63.1 (49.9-72.3)
Males (%)	2912 (28.3)	755 (23.0)	2157 (30.8)	13 804 (28.3)
Nordic born (%)	9664 (93.8)	3150 (95.8)	6514 (92.9)	42 908 (88.1)
**Years of education**				
≤ 9	2480 (24.1)	909 (27.6)	1572 (22.4)	11 500 (23.6)
10-12	4258 (41.3)	1400 (42.6)	2858 (40.8)	20 217 (41.5)
≥ 13	3300 (32.0)	905 (27.5)	2396 (34.2)	15 781 (32.4)
Missing	261 (2.5)	74 (2.3)	187 (2.7)	1203 (2.5)
**Pre-existing exposure** *^a^*				
Age at exposure, median (IQR)	61.1 (49.8-70.1)	62.8 (52.0-70.4)	60.3 (48.9-69.8)	62.4 (52.9-70.7)
Thyroid disease	1237 (12.0)	437 (13.3)	800 (11.4)	3818 (7.8)
Graves disease	118 (1.1)	48 (1.5)	70 (1.0)	374 (0.8)
Hashimoto thyroiditis	1137 (11.0)	395 (12.0)	742 (10.6)	3488 (7.2)
**Pre-existing comorbidities (%)**				
IBD	272 (2.6)	89 (2.7)	183 (2.6)	101 (0.2)
Celiac disease	330 (3.2)	107 (3.3)	223 (3.2)	35 (0.1)
Asthma	506 (4.9)	145 (4.4)	361 (5.1)	1563 (3.2)
Psoriasis	393 (3.8)	151 (4.6)	242 (3.5)	814 (1.7)
Rheumatoid arthritis	287 (2.8)	129 (3.9)	158 (2.3)	712 (1.5)
Type 1 diabetes	332 (3.2)	138 (4.2)	194 (2.8)	870 (1.8)
Any of above	1903 (18.5)	671 (20.4)	1232 (17.6)	3829 (7.9)

Abbreviations: CC, collagenous colitis; IBD, inflammatory bowel disease; LC, lymphocytic colitis; MC, microscopic colitis.

^*a*^Sum of numbers of Graves disease and Hashimoto thyroiditis can be greater than the number with thyroid disease because patients with Hashimoto may convert to Graves disease later on.

Among the population analysis sample, 59.6% subjects (8540 MC cases and 26 618 controls) had at least 1 hospital visit to an inpatient or specialist outpatient clinic during the pre-existing time window (Table S3 (27); note that primary care visits are not recorded in the NPR). The first, second, and third quantile of the number of hospital visits was 2, 4, and 8 (visits), respectively. As expected, patients with MC visited hospital (mean = 8.2; median = 5) more often than controls (mean = 5.7; median = 3) during the 3 years to 6 months prior to index date.

### Characteristics of Sibling Analysis Sample

The sibling-analysis sample comprised 5810 MC cases and 11 210 full siblings who on average were about 4 years younger than the population analysis sample (patients with MC and their controls) (Fig. 1 and Table 2). Contrast between cases and controls was comparable to that in the population sample, except that the sex ratio in the sibling control group was almost 1:1 (due to no matching). Importantly, a higher pre-existing autoimmune burden was observed in siblings (11.5%) vs population controls (7.9%), which seemed to be mainly attributable to the increased prevalence of inflammatory bowel disease (1.6% in siblings and 0.2% in population controls) and celiac disease (0.9% in siblings and 0.1% in population controls).

**Table 2.** Characteristics of patients with MC diagnosed between 2006 and 2017 and their biopsy-free full sibling comparators

	MC cases	Full sibling controls
Total (%)	5810 (100)	11 210 (100)
Age at index date	59.3 (46.0-67.4)	59.0 (47.4-67.0)
Males	1646 (28.3)	5614 (50.1)
Nordic born	5744 (98.9)	11 070 (98.8)
Years of education		
≤9	1044 (18.0)	2278 (20.3)
10-12	2544 (43.8)	5010 (44.7)
≥13	2125 (36.6)	3760 (33.5)
Missing	97 (1.7)	162 (1.4)
Pre-existing exposure		
Age at exposure	56.0 (43.9-62.6)	58.6 (49.6-64.7)
Thyroid disease	629 (10.8)	807 (7.2)
Graves disease*^a^*	69 (1.2)	80 (0.7)
Hashimoto*^a^*	568 (9.8)	739 (6.6)
Pre-existing comorbidities		
IBD	165 (2.8)	176 (1.6)
Celiac disease	230 (4.0)	103 (0.9)
Asthma	277 (4.8)	453 (4.0)
Psoriasis	232 (4.0)	297 (2.6)
Rheumatoid arthritis	144 (2.5)	174 (1.6)
Type 1 diabetes	184 (3.2)	216 (1.9)
Any of above	1097 (18.9)	1290 (11.5)

Values are median (interquartile range) for age variables and N (%) for categorical variables.

Abbreviations: IBD, inflammatory bowel disease; MC, microscopic colitis.

^*a*^Sum of numbers of Graves’ disease and Hashimoto can be greater than the number of thyroid disease because patients with Hashimoto may convert to Graves disease later on.

### Overall Association of MC With Pre-existing AITDs

**Figure 2.**
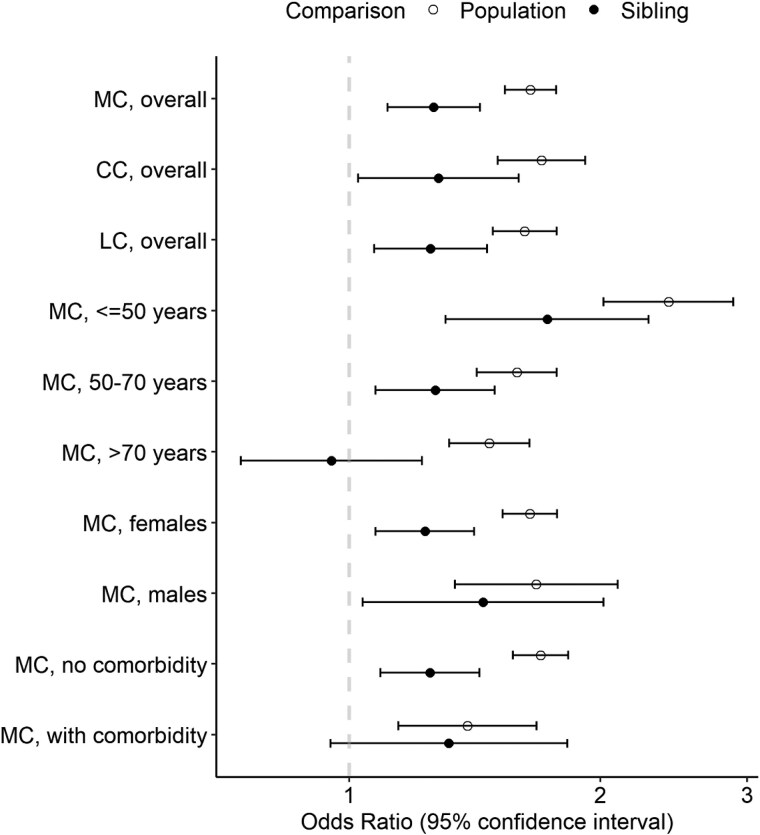
Associations of microscopic colitis and its subtype with prior diagnosis of autoimmune thyroid diseases. CC, collagenous colitis; LC, lymphocytic colitis; MC, microscopic colitis. Subgroups marked as “MC, no comorbidity” and “MC, with comorbidity” denote the subsamples that included participants without any of the selected autoimmune comorbidities (ie, celiac disease, inflammatory bowel disease, asthma, rheumatoid arthritis, type 1 diabetes diagnosis, and psoriasis) and those with at least 1 of the selected comorbidities.

The number of pre-existing diagnoses of AITD was 1237 in MC cases and 3818 in population controls, yielding a crude OR of 1.60 (95% CI 1.50-1.72). After adjusting for matching and other covariates, MC associated significantly with prevalent AITD at an OR of 1.65 (95% CI 1.54-1.77) in the main analysis comparing population controls (Table 3 and Fig. 2). This positive association attenuated but remained statistically significant in our sibling results (OR 1.26; 95% CI 1.11-1.43) and the sensitivity analysis further considering health care–seeking behaviors before the index date (OR 1.40; 95% CI 1.29-1.52) (Table S3 (27)).

**Table 3.** Odds ratios and 95% CIs of associations between autoimmune thyroid disease and subsequent diagnosis of microscopic colitis

	Population controls*^a^*		Sibling controls*^b^*	
	OR (95% CI)	*P* value	OR (95% CI)	*P* value
Overall	1.65 (1.54, 1.77)	3.3 × 10^−43^	1.26 (1.11, 1.43)	3.2 × 10^−4^
By disease subtype				
Collagenous colitis	1.70 (1.51, 1.92)	6.7 × 10^−18^	1.28 (1.03, 1.60)	.03
Lymphocytic colitis	1.62 (1.49, 1.77)	5.0 × 10^−27^	1.25 (1.07, 1.46)	4.7 × 10^−3^
By age at index date				
<50 years	2.41 (2.02, 2.89)	7.0 × 10^−22^	1.73 (1.31, 2.29)	1.3 × 10^−4^
50-70 years	1.59 (1.42, 1.77)	1.4 × 10^−16^	1.27 (1.08, 1.49)	4.8 × 10^−3^
>70 years	1.47 (1.32, 1.64)	6.6 × 10^−12^	0.95 (0.74, 1.22)	.70
By sex				
Females	1.65 (1.53, 1.78)	9.2 × 10^−39^	1.23 (1.08, 1.41)	2.5 × 10^−3^
Males	1.68 (1.34, 2.10)	6.6 × 10^−6^	1.45 (1.04, 2.02)	.03
By number of pre-existing autoimmune comorbidities				
0	1.70 (1.57, 1.83)	2.5 × 10^−42^	1.25 (1.09, 1.43)	1.4 × 10^−3^
≥1	1.39 (1.15, 1.68)	7.8 × 10^−4^	1.32 (0.95, 1.83)	.10

^*a*^Main analysis adjusting for matching group, birth country, education attainment, and the number of pre-existing diagnosis of 6 autoimmune comorbidities.

^*b*^Sensitivity analysis adjusting for family cluster, sex, age at index date, education attainment, and the number of pre-existing diagnosis of 6 autoimmune comorbidities.

### Association of MC With Pre-existing AITD by Subgroup

No difference in association was found across MC subtypes (OR and 95% CI 1.70, 1.51-1.92 for CC and 1.62, 1.49-1.77 for LC) or between males (OR 1.68; 95% CI 1.34-2.10) and females (OR 1.65; 95% CI 1.53-1.78) (Table 3 and Fig. 2). This was also supported by the sibling analysis results. Yet, the association appeared stronger among individuals aged below 50 years at the index date (OR 2.41; 95% CI 2.02-2.89) than those aged 50 or older (OR and 95% CI 1.59, 1.42-1.77 if between 50 and 70 years and 1.47, 1.32-1.64 if older than 70 years). Particularly among those older than 70 years, odds of AITDs were no longer different in sibling comparison analysis (OR 0.95; 95% CI 0.74-1.22).

Stratifying by presence of autoimmune comorbidity the association attenuated somewhat in those with at least 1 autoimmune comorbidity (OR 1.39; 95% CI 1.15-1.68) compared with patients without such disease (OR 1.70; 95% CI 1.57-1.83).

Although the subgroup-level findings were replicated by the sensitivity analysis adjusting for pre-existing hospital visits, the difference by history of autoimmune comorbidities was not seen in sibling analysis (Table 3).

## Discussion

In this nationwide population-based matched case–control study of more than 10 000 patients with MC, we demonstrate that prior AITDs are significantly more common among patients with MC than in controls selected from the general population. Our finding was robust in sibling-controlled analysis and other sensitivity analyses, indicating that the association is not driven by residual confounding including shared genetics and early environmental factors. The association was stronger in patients diagnosed with MC below the age of 50.

### A Comparison With the Literature

To the best of our knowledge, the association between AITDs and MC has not yet been comprehensively explored. A cross-sectional study (12) of 131 women with MC in southern Sweden found that 17.6% of them were treated with thyroid hormones. Another study from the same group examined 133 female patients with MC from the same region and found an OR of 2.98 (95% CI 1.78-4.99) for prevalent thyroid disorders. Nevertheless, temporality was not appropriately considered in either of the 2 studies; the results are thus less informative about the role of AITD as a risk factor or predictor of MC. Besides, a more recent nationwide case–control study in Denmark assessed a total of 42 autoimmune disorders in relation to MC and found an OR of 1.25 (95% CI 1.10-1.42) for GD and 1.43 (95% CI 1.06-1.91) for HT. However, the analysis did not correct for multiple testing and no stratified analyses were implemented either. Hence, despite the growing data linking AITD to MC, there remains a need for carefully designed studies to better understand this relationship.

We assessed the association between AITDs and MC across various strata. Generally, we detected no subgroup differences except for the age at MC diagnosis, where patients diagnosed before age 50 had significantly higher odds of AITD than those diagnosed at more advanced ages. Moreover, our findings were robust across several sensitivity analyses. First, to control for surveillance bias and potentially delayed diagnoses of AITDs, an analysis excluding all study participants with a follow up less than 1 year was conducted. This restriction, however, had no meaningful impact on our point estimate. Finally, to control for intrafamilial confounding, we used full siblings of our patients with MC as controls, yielding a somewhat attenuated aOR.

We also observed similar estimates for CC and LC. Although previous studies have reported different genetic profiles underlying the 2 disease subtypes (30, 31), our finding of a significant association in the sibling-controlled analyses, as well as the comparable aORs for LC and CC, may indicate that shared inflammatory characteristics and an autoimmune predisposition, rather than genetic factors, are the main drivers of our observed association.

### Biologic Mechanisms

Our findings have biologic plausibility. The inflammatory activity in both MC and AITDs are characterized by cytokines in the TH-17 (16-18) pathway. Moreover, previous findings of patients with HT having an increased count of intraepithelial lymphocytes in their colonic mucosa (19), further strengthen the credibility of our observed association. Finally, MC and AITDs have both been linked to the same alleles (32, 33) related to the HLA system.

### Strengths and Weaknesses

This study has several strengths. First, the nationwide scope of the ESPRESSO study (25) minimizes the impact of selection bias. Moreover, the large cohort size improves power of stratified analysis. Additionally, our prior validation work (15) enhances the credibility of our definition of MC. In the same study, we also noted that 96% of patients with MC had a complaint of diarrhea, which confirms the accuracy of our biopsy-based definition. The use of personal identity numbers (21) allowed for effective linkage to multiple registers, enabling the creation of a large and comprehensive cohort of cases and controls. Moreover, the ability to identify full siblings provided a unique opportunity to address intrafamilial confounding.

There are also limitations. Our study was strictly register based, resulting in a lack of information on potentially confounding lifestyle factors such as body mass index (BMI) and smoking. However, as MC is associated with lower BMI (34) and HT (35) (the more common exposure) is associated with higher BMI, additional adjustment for BMI would have strengthened our result. Furthermore, the NPR does not include data on diagnoses from primary care. Nevertheless, as GD almost exclusively managed (at least initially) in specialist care and patients with HT are identifiable from the PDR due to thyroid hormone prescriptions, impact from this issue on analysis might be low. In an effort to control for surveillance bias stemming from the regular check-ups associated with AITDs, we adjusted for pre-existing hospital visits as a proxy for health care–seeking behavior. However, we acknowledge the possibility of residual surveillance bias due to increased monitoring of patients with AITD. While the diagnostic evaluation for MC typically requires specific symptoms (chronic watery diarrhea) to prompt a colonoscopy with biopsy, yearly check-ups for AITD could still facilitate the evaluation of less severe gastrointestinal symptoms. Importantly, primary care visits—where such evaluations may occur—are not captured in the NPR, which could further contribute to residual bias.

Lastly, the inclusion of only Swedish individuals may limit the generalizability of our finding. Consequently, our results may not be directly applicable to populations in other countries with different lifestyle factors, comorbidities, and ethnicities.

To conclude, patients diagnosed with AITDs are at an increased risk of developing MC. The risk was comparable for both LC and CC and both HT and GD were independently associated with MC. Our findings hold clinical relevance for both endocrinologists and general practitioners caring for patients with AITDs. Patients with persistent gastrointestinal symptoms despite achieving euthyroidism should be evaluated for underlying MC. Moreover, our findings contribute scientifically by strengthening the notion that these conditions may share underlying causes as well as underscoring the link between autoimmune conditions and MC.

## Data Availability

Restrictions apply to the availability of some or all data generated or analyzed during this study to preserve patient confidentiality or because they were used under license. The corresponding author will on request detail the restrictions and any conditions under which access to some data may be provided.

## Abbreviations

AITD: autoimmune thyroid disease
BMI: body mass index
CC: collagenous colitis
GD: Graves disease
HT: Hashimoto thyroiditis
LC: lymphocytic colitis
MC: microscopic colitis
NPR: National Patient Register
PDR: Prescribed Drug Register
